# Circ_0082182 promotes oncogenesis and metastasis of colorectal cancer in vitro and in vivo by sponging miR-411 and miR-1205 to activate the Wnt/β-catenin pathway

**DOI:** 10.1186/s12957-021-02164-y

**Published:** 2021-02-17

**Authors:** Ruijie Liu, Ping Deng, Yonglian Zhang, Yonglan Wang, Cuiping Peng

**Affiliations:** 1 Department of General Surgery, Jingmen No.1 People’s Hospital, Jingmen, 448000 Hubei China; 2 Department of Anorectal Surgery, Jingmen No.1 People’s Hospital, No.167, Xiangshan Avenue, Dadao District, Jingmen, 448000 Hubei China; 3 Department of Digestive Endoscopy Center, Jingmen No.1 People’s Hospital, Jingmen, 448000 Hubei China

**Keywords:** circ_0082182, Colorectal cancer, miR-411, miR-1205, Wnt/β-catenin pathway

## Abstract

**Background:**

Circular RNAs (circRNAs) are a class of endogenous single-strand RNA transcripts with crucial regulation in human cancers. The objective of this study is to investigate the role of circ_0082182 in CRC and its specific functional mechanism.

**Methods:**

The quantitative real-time polymerase chain reaction (qRT-PCR) was performed to measure the levels of circ_0082182, microRNA-411 (miR-411) and microRNA-1205 (miR-1205). Cell proliferation was detected by Cell counting Kit-8 (CCK-8) and colony formation assays. Flow cytometry was used for determining cell cycle and cell apoptosis. Cell apoptosis was also assessed by caspase3 and caspase9 activities. Cell migration and invasion were examined using scratch assay and transwell assay. The interaction between circ_0082182 and miRNA was validated by the dual-luciferase reporter and biotinylated RNA pull-down assays. Wnt/β-catenin pathway and epithelial-mesenchymal transition (EMT)-associated proteins were quantified by Western blot. Xenograft model was established for the research of circ_0082182 in vivo.

**Results:**

Circ_0082182 was upregulated in CRC and could predict the poor prognosis of CRC patients. Functionally, circ_0082182 promoted CRC cell proliferation, cell cycle progression, and metastasis while inhibited apoptosis. Subsequently, circ_0082182 was shown to act as the sponges of miR-411 and miR-1205. MiR-411 and miR-1205 were identified as tumor inhibitors in CRC. Furthermore, circ_0082182 promoted the CRC progression via sponging miR-411 and miR-1205. Moreover, circ_0082182 facilitated the Wnt/β-catenin pathway and EMT process by targeting miR-411 and miR-1205. In vivo, circ_0082182 accelerated the CRC tumorigenesis and EMT process by activating the Wnt/β-catenin pathway by downregulating the expression of miR-411 or miR-1205.

**Conclusion:**

This study showed that circ_0082182 functioned as an oncogene in the developing process of CRC by sponging miR-411 or miR-1205 to activate the Wnt/β-catenin pathway. Circ_0082182 might be a molecular target in the diagnosis and treatment of CRC.

**Supplementary Information:**

The online version contains supplementary material available at 10.1186/s12957-021-02164-y.

## Introduction

Colorectal cancer (CRC) that affects colon and rectum is one of the leading causes of human death from cancers [1]. The current global cancer statistics have indicated that over 1.8 million new CRC cases and 881,000 deaths were occurred in 2019 [2]. Increasing therapies (local surgical excision, preoperative radiotherapy, palliative chemotherapy, and immunotherapy) have been developed for the locoregional and metastatic CRC, but the survival remains quite poor [3]. Genetic alterations have important influences on contributing to the pathological development, and the molecular markers can be used as the therapeutic targets for CRC [4]. It is essential to seek more effective biomarkers. Non-coding RNAs (ncRNAs) are vital regulators in the occurrence and metastasis of cancers by interacting with cell signaling pathways [5].

Circular RNAs (circRNAs) are usually found in the cytoplasm of eukaryotic cells with the circular forms [6]. CircRNAs have been involved in tumor response of many malignancies [7], such as oral cancer [8], osteosarcoma [9], and nasopharyngeal carcinoma [10]. In addition, circRNAs exhibit the specific biological roles as the sponges of non-coding microRNAs (miRNAs) [11]. For example, circNFIC inhibited cell proliferation and migration in breast cancer by the sponge effect on miR-658 [12]; circCDR1as exerted the oncogenic property in cholangiocarcinoma via serving as a miR-641 sponge [13]. For CRC, circMBOAT2 has been found to promote proliferation/migration of CRC cells by sponging miR-519d-3p [14], and circ_100146 enhanced CRC cell migration/invasion by targeting miR-149 [15]. Chen et al. also clarified that circ-ERBIN contributed to cell growth and metastasis by acting as the sponges of miR-125a-5p and miR-138-5p [16].

Ye et al. have reported that circ_0082182 was upregulated in CRC samples and it might be related to the CRC progression [17]. Recent studies of CRC suggested that microRNA-411 (miR-411) repressed the malignant behaviors of CRC cells [18] and microRNA-1205 (miR-1205) impeded the epithelial-mesenchymal transition (EMT) process [19]. For the sponge effects on miR-411, circ_000926 and circ_001569 have respectively been identified as pro-cancerous factors in renal cell carcinoma and hepatocellular carcinoma by sponging miR-411 [20, 21]. For miR-1205, circ_102958 sponged miR-1205 to regulate the progression of ovarian cancer and circCYFIP2 functioned as a miR-1205 sponge to affect cell metastasis of gastric cancer [22, 23]. In addition to the biological function of circ_0082182 in CRC, its potential sponge effects on miR-411 and miR-1205 were researched in this study.

Wnt/β-catenin pathway is pivotal for embryo development and tissue homeostasis [24], and its activation is implicated in the carcinogenesis of CRC [25]. The previous studies have also found that the regulatory effects of circRNAs and miRNAs on CRC progression were related to the Wnt/β-catenin pathway [26, 27]. Wu et al. reported that miR-1205 could regulate the Wnt/β-catenin pathway in the development of osteosarcoma [28]. This study explored whether circ_0082182 could regulate the Wnt/β-catenin pathway in CRC via affecting miRNA targets, providing a specific pathological mechanism of CRC.

## Materials and methods

### Tissue samples

CRC tissues (*n* = 73) and normal paracancerous tissues (*n* = 73) were obtained during the colorectal resection of 73 CRC patients at Jingmen No.1 People’s Hospital from 2012 to 2015. These tissue samples were saved at − 80 °C for RNA isolation. All cases of patients were extragenetic sporadic CRC. There was no treatment for any of the patients before surgery. The clinicopathological features of CRC patients have been shown in Table 1. The written informed consent forms were signed by 73 patients. This study was approved by the Ethics Committee of Jingmen No.1 People’s Hospital and the procedures were performed in compliance with the Declaration of Helsinki.

**Table 1:** Correlation between the circ_0082182 expression and clinicopathological features of CRC patients

Clinicopathological features	*n*	circ_0082182		*P*
		High (*n* = 32)	Low (*n* = 41)	
Gender				
Female	40	21	19	0.100
Male	33	11	22	
Age (years)				
≥ 60	24	13	11	0.213
< 60	49	19	30	
Tumor size (cm)				
≥ 5	44	24	20	0.023
< 5	29	8	21	
TNM stage				
I–II	34	9	25	0.005
III–IV	39	23	16	
Lymph node metastasis				
Yes	31	18	13	0.035
No	42	14	28	

### Cell culture and transfection

CRC cell lines used in the present study were bought from American Type Culture Collection (ATCC, Manassas, VA, USA): HCT116, SW480, SW620, and CaCo-2. Normal enterocyte NCM460 was purchased from QCHENG BIO (Shanghai, China). Cells were cultivated in a humid environment containing 5% CO_2_ at 37 °C using the homogeneous mixture by Dulbecco’s modified eagle medium (DMEM; Gibco, Carlsbad, CA, USA), 10% fetal bovine serum (FBS; Gibco), and 1% penicillin-streptomycin solution (Gibco).

Short hairpin RNA (shRNA) targeting circ_0082182 (sh-circ) and negative control (sh-NC) vectors, miR-411/miR-1205 mimic (miR-411 and miR-1205) and miRNA mimic NC (miR-NC), miR-411/miR-1205 inhibitor (anti-miR-411 and anti-miR-1205), and miRNA inhibitor NC (anti-NC) were all acquired from GenePharma (Shanghai, China). HCT116 and CaCo-2 cells were transfected with the above products using Lipofectamine™ 3000 Transfection Reagent (Invitrogen, Carlsbad, CA, USA), referring to the manufacturer’s specification.

### Cell groups

HCT116 and CaCo-2 cells were transfected with different groups according to the experimental needs. For the functional assays of circ_0082182, HCT116 and CaCo-2 cells were transfected with sh-NC or sh-circ to construct the stable cell lines (two groups: sh-NC, sh-circ). For the functional analysis of miR-411 and miR-1205, HCT116 and CaCo-2 cells were transfected with miR-NC, miR-411, miR-1205 (three groups: miR-NC, miR-411, miR-1205). For the reverted experiments, shRNA cell lines were transfected with anti-NC, anti-miR-411, or anti-miR-1205 (four groups: sh-NC+anti-NC, sh-circ+anti-NC, sh-circ+anti-miR-411, sh-circ+anti-miR-1205).

### The quantitative real-time polymerase chain reaction

After the extraction of total RNA by TRIzol™ Reagent (Invitrogen), 1000 ng RNA was used for the reverse transcript to synthesize the complementary DNA (cDNA) using Path-ID™ Multiplex One-Step RT-PCR Kit (Applied Biosystems, Foster City, CA, USA) and TaqMan™ MicroRNA Reverse Transcription Kit (Applied Biosystems). The expression determination was performed by TaqPath™ qPCR Master Mix, CG (Applied Biosystems) and the ABI7500 Fast System (Applied Biosystems). The 2^-∆∆Ct^ method was applied to analyze the relative levels of circ_0082182 (normalized by glyceraldehyde-phosphate dehydrogenase, GAPDH) and miR-411/miR-1205 (normalized by U6). The primer sequences used for qPCR were listed in Table 2.

**Table 2:** Primer sequences used for qRT-PCR

Name	Primer sequences
circ_0082182	Forward: 5′-GGAGCCTGACACCTAGGCAA-3′ Reverse: 5′-GGGTGTTTTTCGTGGAGCTT-3′
miR-411	Forward: 5′-GGGGTAGTAGACCGTATAG-3′ Reverse: 5′-TGCGTGTCGTGGAGTC-3′
miR-1205	Forward: 5′-CTGCAGGGTTTGCTTTGAGG-3′ Reverse: 5′-CTCCAGAACAGGGTTGACAGG-3′
GAPDH	Forward: 5′-GACAAGCTTCCCGTTCTCAG-3′ Reverse: 5′-GAGTCAACGGATTTGGTCGT-3′
U6	Forward: 5′-CTCGCTTCGGCAGCACA-3′ Reverse: 5′-AACGCTTCACGAATTTGCG-3′

### Cell counting Kit-8 () assay

2 × 10^3^ HCT116 and CaCo-2 cells (100 μL) were plated into the 96-well plates for 24 h. After different transfection for different times (0 h, 24 h, 48 h and 72 h), 10 μL Cell counting Kit-8 (CCK-8) solution (Beyotime, Shanghai, China) was pipetted into the well to incubate cells for 2 h. Cell absorbance was measured at 450 nm using a microplate reader (Bio-Rad, Hercules, CA, USA).

### Colony formation assay

Two hundred cells in the 6-well plates were cultured in the 37 °C incubator for 2 weeks until the colonies were observed. These colonies were fixed and stained using 4% Paraformaldehyde Fix Solution and Crystal Violet Staining Solution (Beyotime), followed by the manual counting of colonies.

### Flow cytometry for cell cycle and apoptosis

Tali® Cell Cycle Kit (Invitrogen) was used for cell cycle detection. Briefly, 4 × 10^6^ cells were washed by Dulbecco’s phosphate-buffered saline (DPBS; Gibco) and cells were fixated by ice-cold 70% ethanol (Sigma-Aldrich, St. Louis, MO, USA) in distilled water at − 20 °C overnight. In the dark, cells were stained with Tali® Cell Cycle Solution that was composed of propidium iodide (PI), RNase A, and Triton X-100 for 30 min. Eventually, cell proportion of each phase (G0/G1, S, and G2/M) was respectively determined using the flow cytometer (BD Biosciences, San Diego, CA, USA).

Cell apoptosis was detected by ApoDETECT Annexin V-FITC Kit (Invitrogen). Cell density was adjusted to 4 × 10^5^ cells/mL in 1× binding buffer, and 10 μL Annexin V-FITC was added to 190 μL cell suspension. After incubation for 10 min at room temperature, cells were incubated with 10 μL PI stock solution. Cell analysis was performed by a flow cytometer (BD Biosciences). The apoptotic rate (%) was calculated using the ratio of apoptotic cells and total cells.

### Caspase3 and caspase9 activity detection

At 72 h post-transfection, cells were harvested by centrifugation at 1000 g for 5 min. Then caspase3 and caspase9 activities in 2 × 10^6^ cells were examined using caspase3 colorimetric assay kit and caspase9 activity test kit (Solarbio, Beijing, China) as per the user’s manuals.

### Scratch assay

HCT116 and CaCo-2 cells were seeded into the 6-well plates. Two vertical scratches were scraped by a sterile pipette tip in single-layer cells. The waste cells were removed by PBS (Gibco), and cell culture was performed in DMEM + 10% FBS for 24 h. Cells were photographed at 0 h and 24 h under a microscope (× 40 magnification). The scratch width was measured and the migration rate (%) was calculated using the following formula: (scratch width at 0 h − scratch width at 24 h)/scratch width at 0 h.

### Transwell invasion assay

1 × 10^5^ HCT116 and CaCo-2 cells were suspended in serum-free medium, then inoculated into the top chamber of transwell chamber (Corning Inc., Corning, NY, USA) pre-coated with matrigel (Corning Inc.). The bottom chamber was added with DMEM medium containing 10% FBS. After 24 h, cells passed into the bottom chamber were fastened in 4% paraformaldehyde fix solution (Beyotime) and dyed using Crystal Violet Staining Solution (Beyotime). The pictures of invasive cells were acquired by an inverted microscope (× 100 magnification; Olympus, Tokyo, Japan) and cell number was counted in the arbitrary three fields.

### Dual-luciferase reporter assay

Circinteractome (https://circinteractome.nia.nih.gov) was used for bioinformatics analysis. The circ_0082182 sequence (wild-type, WT) and its mutant sequence (mutant-type, MUT) targeting the miR-411 binding sites were respectively cloned into the pmirGLO vector (Promega, Madison, WI, USA). The novel plasmids were named as circ_0082182 WT1 and circ_0082182 MUT1. The circ_0082182 luciferase plasmids targeting miR-1205 (circ_0082182 WT2 and circ_0082182 MUT2) were constructed in a similar way. After co-transfection of each circ_0082182 plasmid and miRNA mimic or miR-NC for 48 h, the firefly and renilla luciferase activities in HCT116 and CaCo-2 cells were examined using the dual-luciferase reporter assay system (Promega) following the supplied operating steps. Finally, the relative luciferase activity (firefly/renilla) was calculated.

### Biotin-coupled miRNA capture

Biotin-coupled miRNA mimics (GenePharma) for miR-411 (Bio-miR-411 and Bio-miR-411-MUT) and miR-1205 (Bio-miR-1205 and Bio-miR-1205-MUT) were respectively transfected into HCT116 and CaCo-2 cells, with Bio-miR-NC as the negative control. Whereafter, cells were lysed and incubated with Pierce™ Streptavidin Magnetic Beads (Thermo Fisher Scientific, Waltham, MA, USA) at 4 °C overnight. Total RNA was purified from the magnetic beads and circ_0082182 enrichment was assayed by quantitative real-time polymerase chain reaction (qRT-PCR).

### Western blot

RIPA Lysis and Extraction Buffer (Thermo Fisher Scientific) was applied for protein extraction from cells and tissues. The lysates were then mixed with protein loading buffer (Beyotime) and denatured in the boiling water bath for 10 min. The protein products were immediately used for the expression determination as previously described [29]. Antibodies were purchased from Cell Signaling Technology (CST, Boston, MA, USA): phosphorylated (Ser 9) glycogen synthase kinase 3 beta (p-GSK3β; #5558, 1:1000), GSK3β (#12456, 1:1000), β-catenin (#8480, 1:1000), E-cadherin (#3195, 1:1000), N-cadherin (#13116, 1:1000), reference gene GAPDH (#5174, 1:1000), and Anti-rabbit IgG, HRP-linked second antibody (#7074, 1:3000). Protein blotting could be emerged using SignalFire™ Elite ECL Reagent (CST) and protein quantification was performed by ImageLab software version 4.1 (Bio-Rad).

### Animal assay

Ten male BALB/c nude mice (6 weeks old, 20–25 g) were bought from Vital River Laboratory Animal Technology Co., Ltd. (Beijing, China). Mice were divided into two groups (5 mice/group) and hypodermically injected with 3 × 10^6^ stable HCT116 cells (sh-NC group, sh-circ group). Tumor length (L, mm) and weight (W, mm) were weekly measured by a vernier caliper and tumor volume (mm^3^) was expressed as: L (mm) × W^2^ (mm^2^)/2. After 4 weeks, mice were sacrificed by gradually increasing the concentration of CO_2_ (flow rate, 30% of volume/min) in the sealed container. Tumors were then excised and weighed, followed by the RNA and protein extraction for qRT-PCR and Western blot detection. All animal operations were in accordance with the Animals Guidelines of National Institutes of Health and this assay was ratified by Animal Ethics Committee of Jingmen No.1 People’s Hospital.

### Statistical analysis

Data were shown as the mean ± standard deviation (SD) and statistical analysis was performed by SPSS 24.0. Survival curves were produced by Kaplan-Meier plot and analyzed by log-rank test. Difference analysis was performed using Student’s *t* test for two groups and one-way analysis of variance (ANOVA) followed by Tukey’s test for multiple groups. *P* < 0.05 was deemed as significant difference.

## Results

### Circ_0082182 was upregulated in CRC and associated with a poor prognosis

**Fig. 1:**
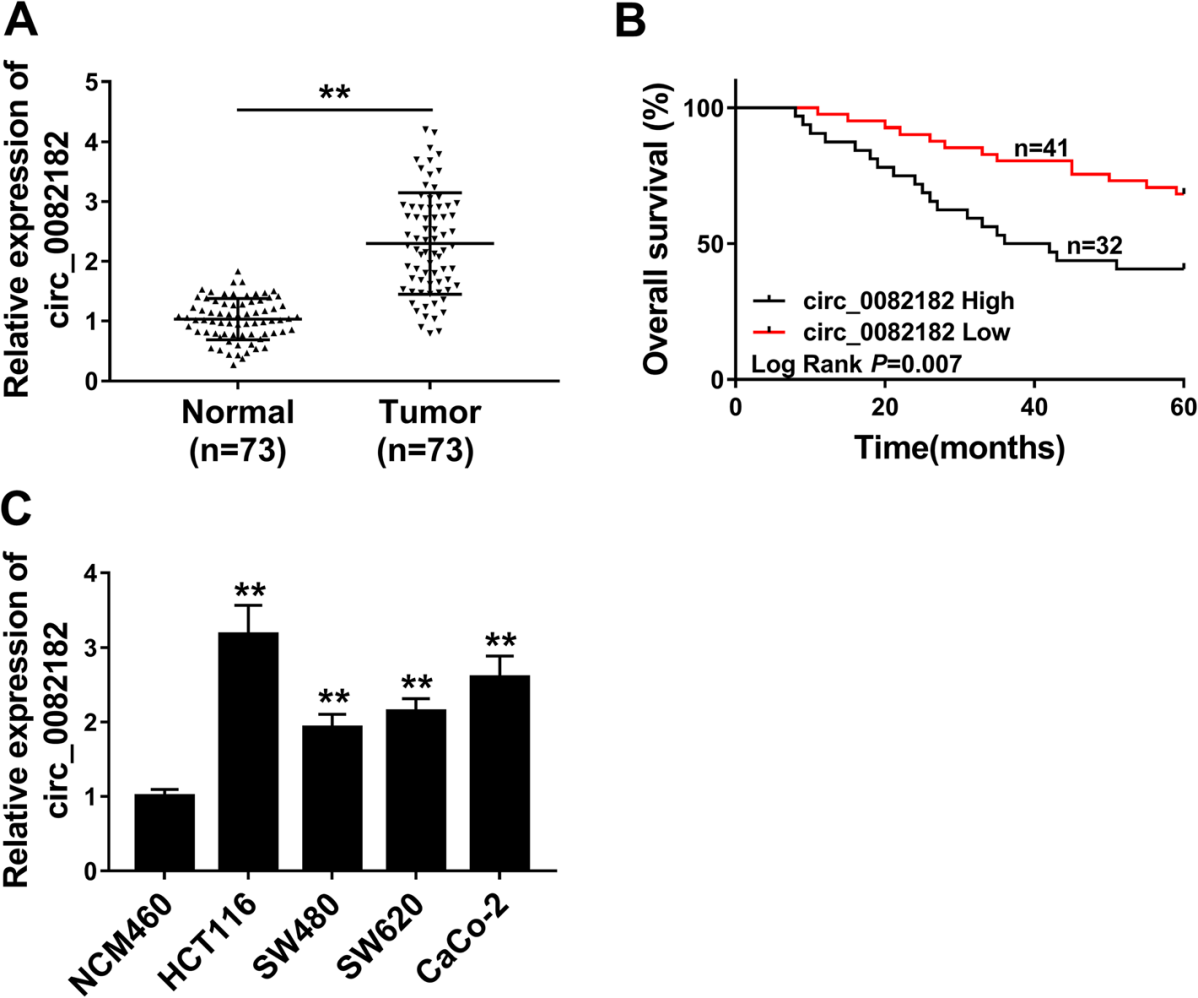
Circ_0082182 was upregulated in CRC and associated with a poor prognosis. **a** Circ_0082182 was upregulated in CRC tissues compared with normal tissues by qRT-PCR analysis. **b** High circ_0082182 expression could predict the poor survival of CRC patients. **c** Circ_0082182 level was higher in CRC (HCT116, SW480, SW620 and CaCo-2) cells than that in normal NCM-460 cells. **P < 0.01

Circ_0082182 was overexpressed in CRC samples by the previous research [17]. Consistently, our qRT-PCR analysis indicated that circ_0082182 level was markedly increased in 73 CRC samples by contrast with 73 normal counterparts (Fig. 1a). The 5-year survival analysis manifested that the overall survival rate was lower in CRC patients with high circ_0082182 expression (*n* = 32) than those patients with low circ_0082182 expression (*n* = 41) (Fig. 1b). Also, the clinicopathological features of CRC patients demonstrated that the high expression of circ_0082182 was associated with TNM stage (*P* = 0.005) (Table 1), showing that circ_0082182 could aggravate the malignant progression of CRC patients. In addition, the obvious upregulation of circ_0082182 was found in four CRC cell lines (HCT116, SW480, SW620, and CaCo-2) compared with the normal NCM460 cell line (Fig. 1c). HCT116 and CaCo-2 cells with higher circ_0082182 expression (relative to SW480 and SW620) were chosen for the following assays. Therefore, circ_0082182 might be related to CRC progression and it had crucial prognostic value for the clinical patients.

### Circ_0082182 contributed to cell proliferation and cell cycle progression while reduced apoptosis in CRC cells

**Fig. 2:**
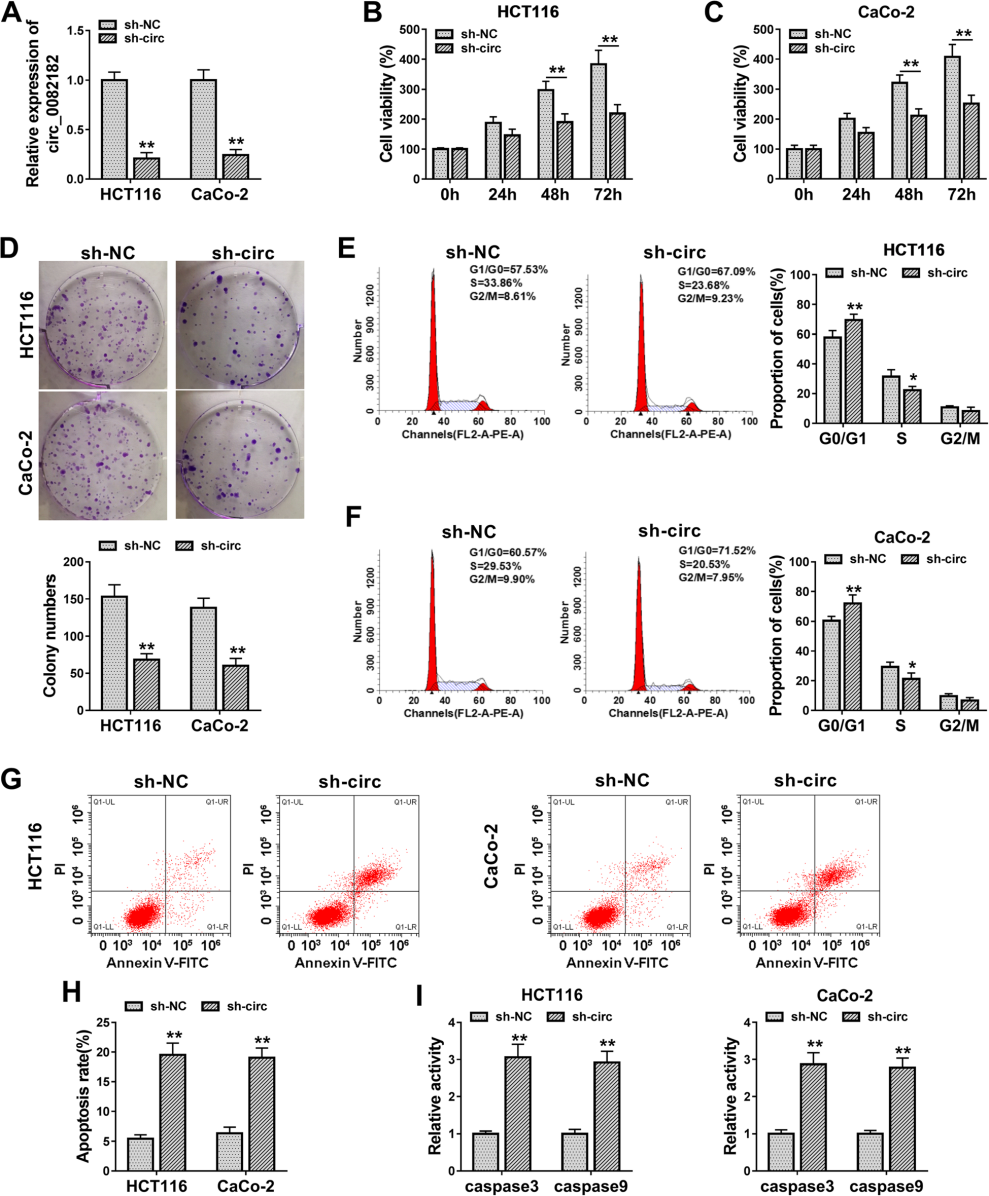
Circ_0082182 contributed to cell proliferation and cell cycle progression while reduced apoptosis in CRC cells. **a** Circ_0082182 expression was knocked down by sh-circ in HCT116 and CaCo-2 cells. **b**–**d** Cell proliferation by CCK-8 assay (**b**, **c**) and colony formation (**d**) assay was inhibited after circ_0082182 was downregulated. **e**, **f** Cell cycle progression by flow cytometry was blocked by the knockdown of circ_0082182. **g, i** Cell apoptotic rate (**g**, **h**) and caspase3/caspase9 activities (**i**) were increased in sh-circ group relative to sh-NC group. *P < 0.05, **P < 0.01.

To explore the regulatory function of circ_0082182 in CRC progression, circ_0082182 expression was knocked down in HCT116 and CaCo-2 cells by establishing the sh-NC/sh-circ-transfected stable cell lines. As shown in Fig. 2a, the expression of circ_0082182 was evidently reduced in sh-circ group relative to sh-NC group. Cell proliferation was analyzed by CCK-8 assay (Fig. 2b, c) and colony formation assay (Fig. 2d), and the results suggested that cell proliferative ability was inhibited in sh-circ-transfected cells contrasted with sh-NC-transfected cells. Cell cycle analysis revealed that cell transition was blocked from G0/G1 phase to S phase after the knockdown of circ_0082182 (Fig. 2e, f). Flow cytometry also showed that cell apoptotic rate was much higher in sh-circ group than that in sh-NC group (Fig. 2g, h). Meanwhile, the downregulation of circ_0082182 enhanced the activities of pro-apoptotic caspase3 and caspase9 in HCT116 and CaCo-2 cells (Fig. 2i). The above evidence confirmed the oncogenic role of circ_0082182 in the development of CRC.

### Circ_0082182 functioned as a metastasis-promoting factor in CRC cells

**Fig. 3:**
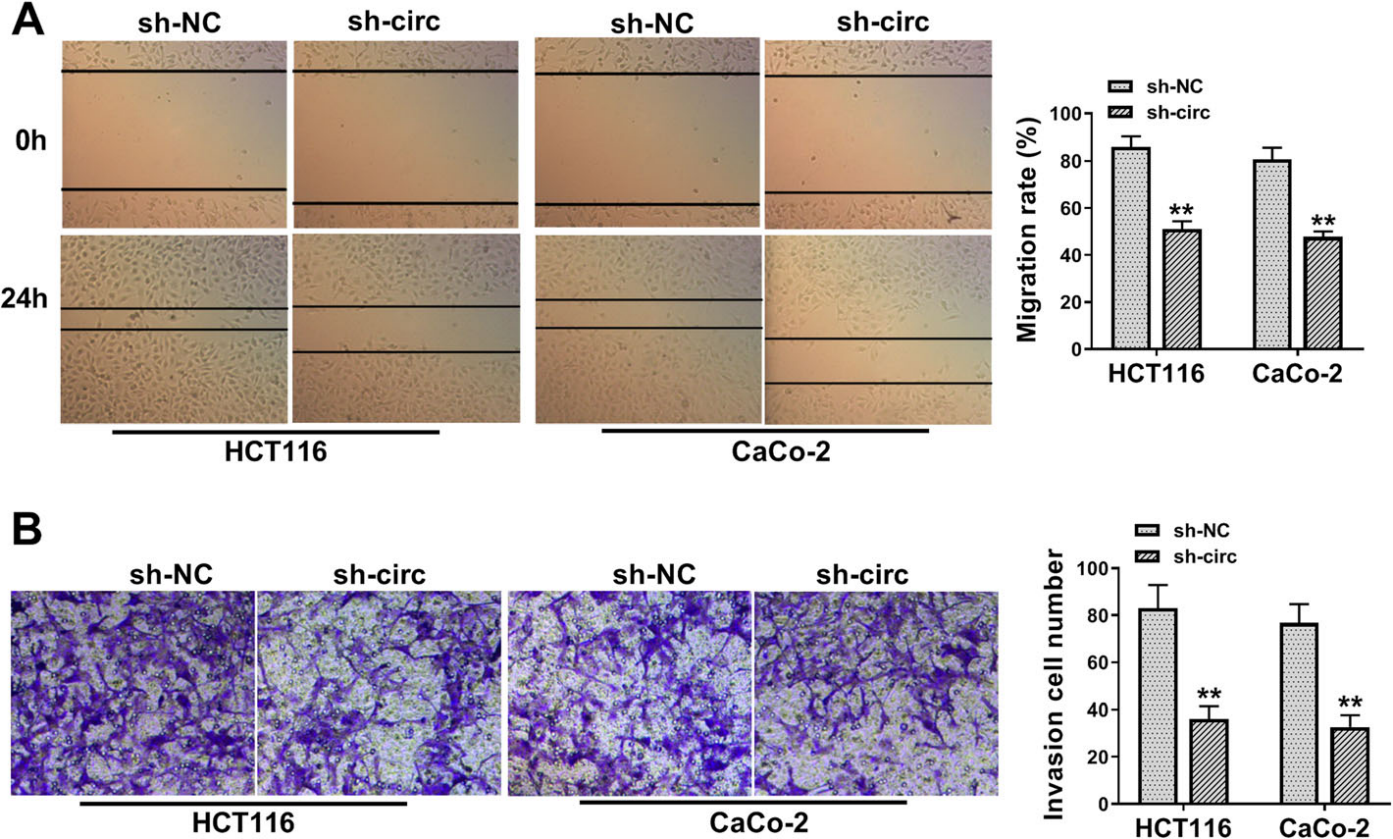
Circ_0082182 functioned as a metastasis-promoting factor in CRC cells. **a**, **b** Downregulation of circ_0082182 reduced the cell migratory rate in the scratch assay (**a**) and the invaded cell number in the transwell assay (**b**). **P < 0.01

CircRNAs have been reported to participate in tumor metastasis, including CRC [7]. The clinicopathological features in Table 1 also indicated that circ_0082182 was correlated to lymph node metastasis of CRC patients (*P* = 0.035). To analyze the effect of circ_0082182 on CRC cell metastasis, cell migration and invasion were respectively detected using scratch assay and transwell assay. As Fig. 3a, b depicted, the silence of circ_0082182 suppressed the migratory rate and invasive cell number of HCT116 and CaCo-2 cells. Thus, circ_0082182 promoted the metastasis of CRC cells.

### Circ_0082182 targeted miR-411 and miR-1205 in CRC cells

**Fig. 4:**
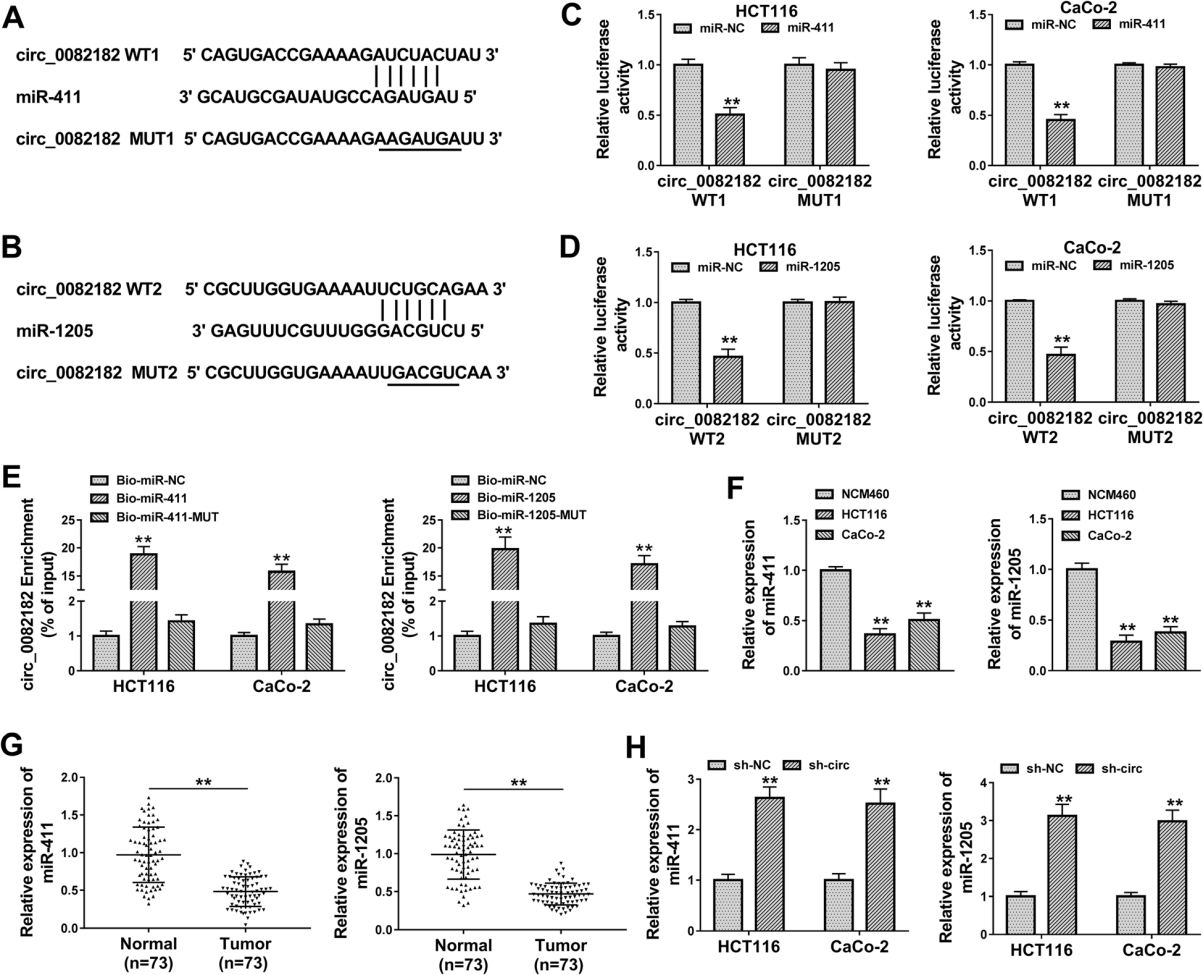
Circ_0082182 targeted miR-411 and miR-1205 in CRC cells. **a**, **b** Circinteractome showed the binding sites between circ_0082182 and miR-411 (**a**) or miR-1205 (**b**). **c, e** The interaction between circ_0082182 and miR-411 or miR-1205 was validated using the dual-luciferase reporter assay (**c**, **d**) and Bio-coupled RNA pull-down assay (**e**). **f, g** The expression levels of miR-411 and miR-1205 were downregulated in CRC cells (**f**) and tissues (**g**). **h** Knockdown of circ_0082182 induced the upregulation of miR-411 and miR-1205 in HCT116 and CaCo-2 cells. **P < 0.01

CircRNAs usually have sponge effects on miRNAs in cancer regulation [30]. Online circinteractome predicted the binding sites between the sequences of circ_0082182 and miR-411 (Fig. 4a) or miR-1205 (Fig. 4b), exhibiting that miR-411 and miR-1205 might be the targets for circ_0082182. By performing the dual-luciferase reporter assay, we found that miR-411 (Fig. 4c) or miR-1205 (Fig. 4d) overexpression respectively repressed the luciferase signals of circ_0082182 WT1 or circ_0082182 WT2 plasmid but not their MUT plasmids. Moreover, circ_0082182 was largely captured by biotinylated miR-411 and miR-1205 in pull-down assay, in comparison to the negative control group (Bio-miR-NC) and their MUT groups (Fig. 4e). The qRT-PCR showed that miR-411 and miR-1205 levels were downregulated in CRC cells (Fig. 4f) and tissue samples (Fig. 4g). Additionally, knockdown of circ_0082182 induced the stimulative effects on the miR-411 and miR-1205 levels (Fig. 4h). These findings revealed that circ_0082182 could act as the sponges of miR-411 and miR-1205.

### MiR-411 and miR-1205 retarded the progression of CRC

**Fig. 5:**
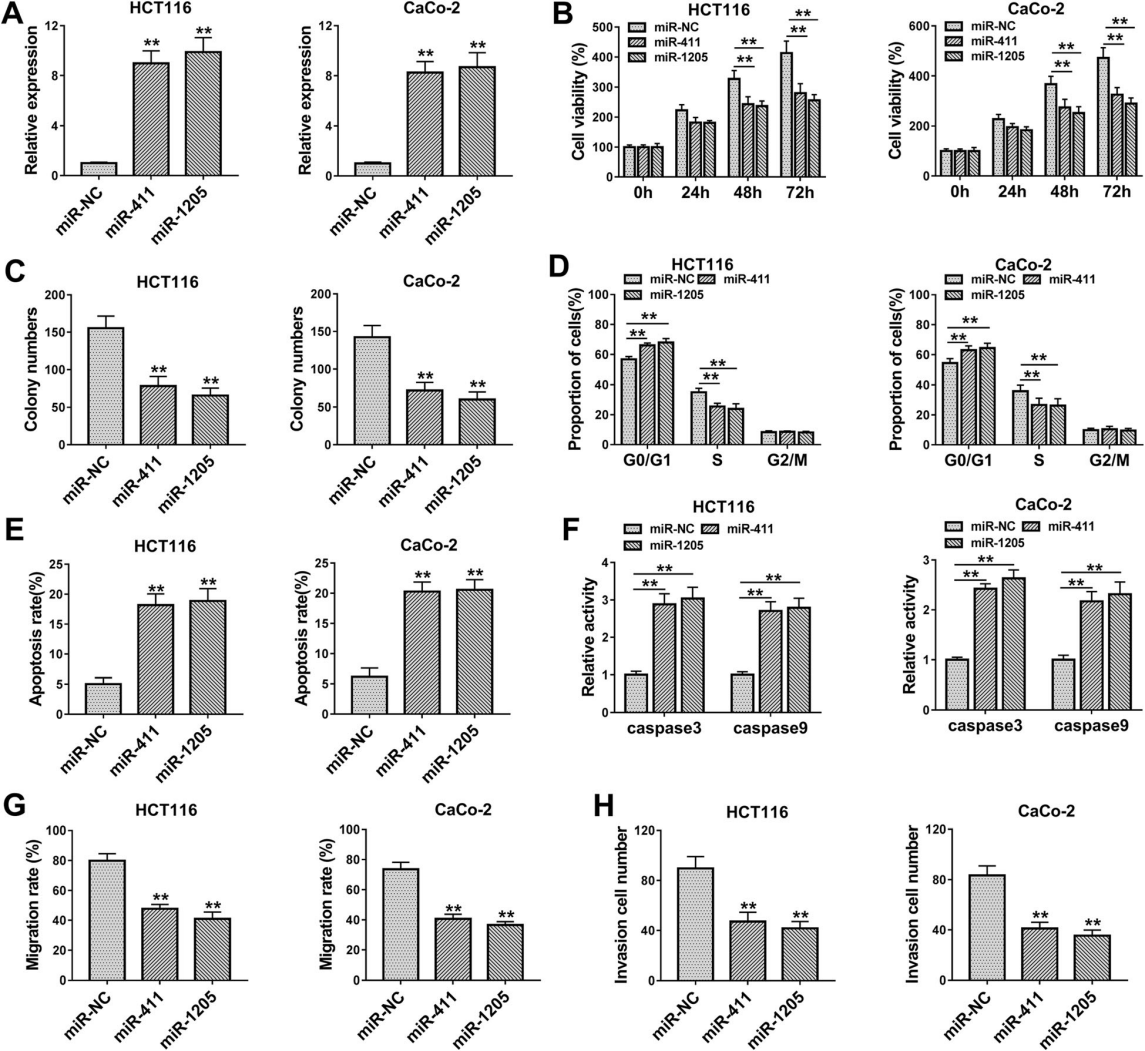
MiR-411 and miR-1205 retarded the progression of CRC. **a** MiR-411 and miR-1205 levels were overexpressed by transfection of miR-411 and miR-1205. **b**–**d** Cell proliferation (**b, c**) and cell cycle (**d**) were suppressed in miR-411 or miR-1205 group contrasted with miR-NC group. **e, f** Transfection of miR-411 or miR-1205 enhanced the apoptotic rate (**e**) and the caspase3/caspase9 activities (**f**). **g**, **h** Overexpression of miR-411 or miR-1205 triggered the inhibition of cell migration (**g**) and invasion (**h**). **P < 0.01

Furthermore, the biological roles of miR-411 and miR-1205 were studied by transfection of miRNA mimics in HCT116 and CaCo-2 cells. The overexpression efficiencies of miR-411 and miR-1205 transfection were shown to be excellent by qRT-PCR (Fig. 5a). Cellular experiments suggested that transfection of miR-411 or miR-1205 resulted in cell viability inhibition (Fig. 5b), colony formation repression (Fig. 5c and Supplementary Fig. 1A), and cell cycle arrest (Fig. 5d and Supplementary Fig. 1B). As for cell apoptosis, the apoptotic rate (Fig. 5e and Supplementary Fig. 1C) and caspase3/caspase9 activities (Fig. 5f) were enhanced by miR-411 or miR-1205 mimic in comparison with miR-NC group. Scratch and transwell assays demonstrated that the overexpression of miR-411 or miR-1205 repressed the abilities of cell migration (Fig. 5g and Supplementary Fig. 1D-E) and invasion (Fig. 5h and Supplementary Fig. 1F). All in all, miR-411 and miR-1205 worked as tumor inhibitors in CRC in vitro.

### Circ_0082182 acted as a carcinogene in CRC by sponging miR-411 and miR-1205

**Fig. 6:**
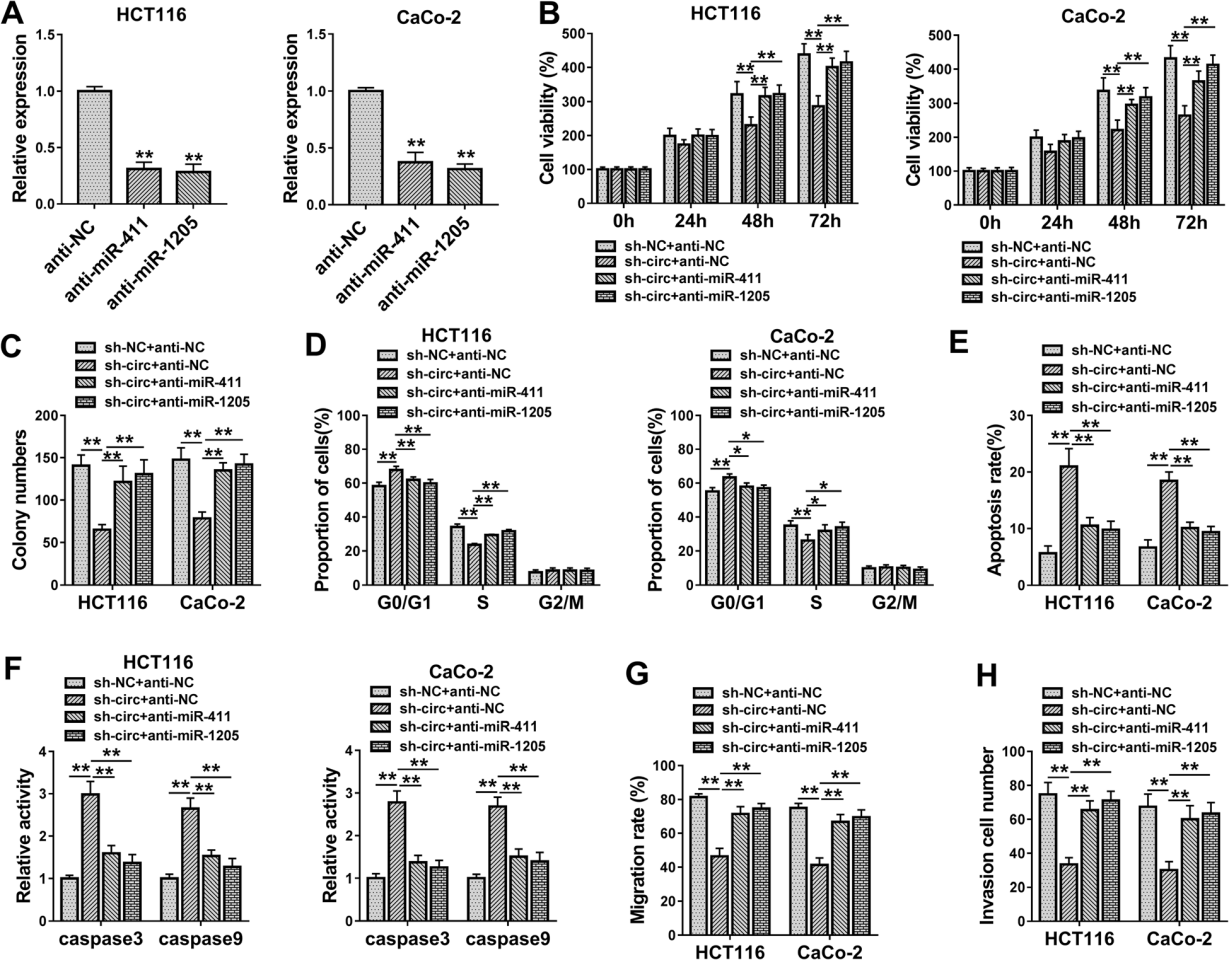
Circ_0082182 acted as a carcinogene in CRC by sponging miR-411 and miR-1205. **a** The inhibitory efficiencies of anti-miR-411 and anti-miR-1205 were shown to be great via qRT-PCR detection. **b**–**d** Inhibitor of miR-411 or miR-1205 eliminated the sh-circ-induced proliferation inhibition (**b**, **c**) and cell cycle arrest (**d**). **e, f** The promoting effects of sh-circ on cell apoptotic rate (**e**) and caspase3/caspase9 activities (**f**) were relieved by anti-miR-411 or anti-miR-1205. **g, h** Sh-circ-mediated inhibition of cell migration (**g**) and invasion (**h**) was restored by miR-411 or miR-1205 inhibitor. *P < 0.05, **P < 0.01

The rescued experiments were conducted to investigate whether the regulation of circ_0082182 was attributed to the sponge effect on miR-411 or miR-1205. The qRT-PCR analysis manifested that the expression inhibition of miR-411 and miR-1205 mediated by anti-miR-411 and anti-miR-1205 was significant (Fig. 6a). Subsequently, anti-miR-411 or anti-miR-1205 transfection was found to counteract the suppressive influences of circ_0082182 knockdown on cell proliferation (Fig. 6b, c and Supplementary Fig. 2A), cell cycle (Fig. 6d and Supplementary Fig. 2B), and the promoting effect on cell apoptosis (Fig. 6e, f and Supplementary Fig. 2C). Additionally, the sh-circ-induced suppression of cell migration (Fig. 6g and Supplementary Fig. 2D-E) and invasion (Fig. 6h and Supplementary Fig. 2F) was also weakened by the downregulation of miR-411 or miR-1205. These data revealed that circ_0082182 promoted cancer progression by sponging miR-411 or miR-1205 in CRC cells.

### Circ_0082182 sponged miR-411 and miR-1205 to activate the Wnt/β-catenin pathway and promote EMT process in CRC cells

**Fig. 7:**
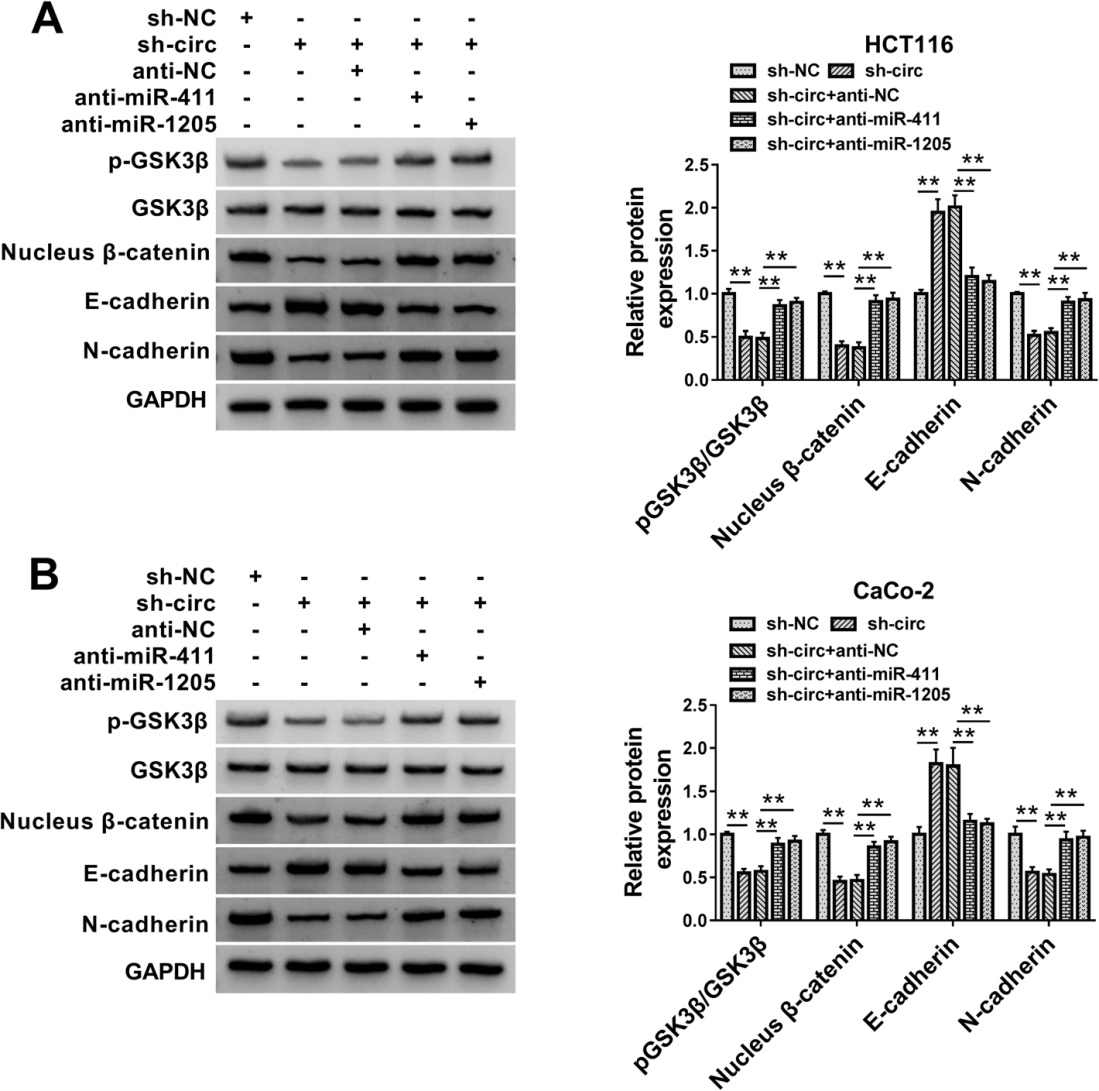
Circ_0082182 sponged miR-411 and miR-1205 to activate Wnt/β-catenin pathway and promote EMT process in CRC cells. **a, b** Knockdown of circ_0082182 repressed the Wnt/β-catenin pathway and EMT process in HCT116 and CaCo-2 cells by upregulating the level of miR-411 or miR-1205. **P < 0.01

Given the involvement of Wnt/β-catenin pathway in CRC progression [25] and the regulation of miR-1205 in the Wnt/β-catenin pathway [28], we further analyzed whether circ_0082182 could affect the Wnt/β-catenin pathway by targeting miR-1205 or miR-411 in CRC cells. Western blot results presented that the protein levels of pGSK3β/GSK3β, Nucleus β-catenin, and N-cadherin were downregulated but E-cadherin was upregulated by the repression of circ_0082182 expression, whereas these effects were mitigated by miR-411 or miR-1205 inhibitor (Fig. 7a, b). Hence, knockdown of circ_0082182 blocked the Wnt/β-catenin pathway and inhibited the EMT process in CRC cells via increasing the miR-411 and miR-1205 expression.

### Circ_0082182 facilitated CRC tumorigenesis and EMT in vivo by activating the miR-411/miR-1205-mediated Wnt/β-catenin pathway

**Fig. 8:**
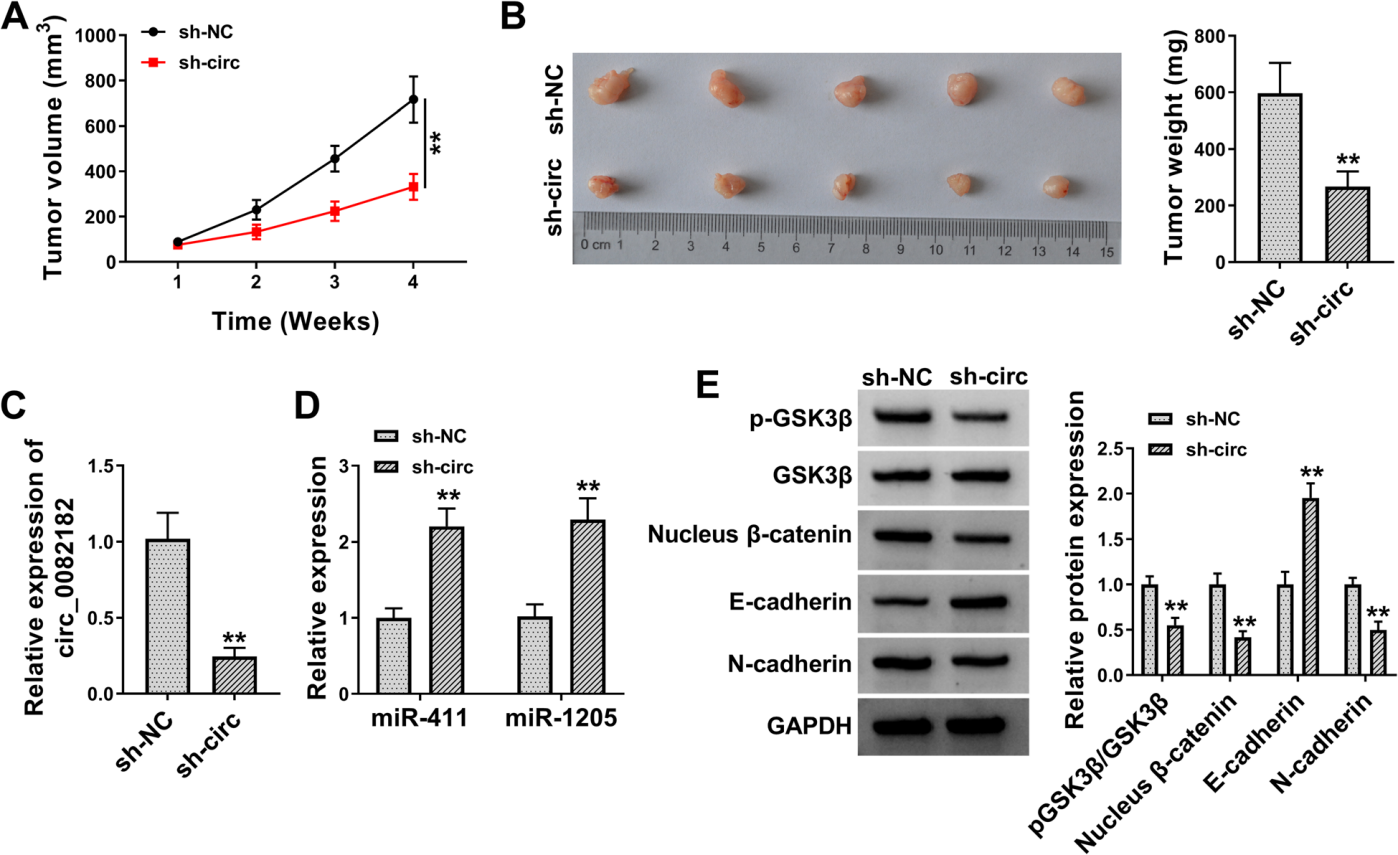
Circ_0082182 facilitated CRC tumorigenesis and EMT in vivo by activating the miR-411/miR-1205-mediated Wnt/β-catenin pathway. **a** Tumor volume was lower in sh-circ group than that in sh-NC group. **b** Tumors were excised and tumor weight was inhibited in sh-circ group by comparison with sh-NC group. **c**, **d** The circ_0082182 expression was downregulated (**c**) and miR-411/miR-1205 levels were increased (**d**) by sh-circ. **e** Silence of circ_0082182 inactivated the Wnt/β-catenin pathway and EMT process in tumor tissues. **P < 0.01

The function of circ_0082182 in vivo was further analyzed by xenograft assay in mice. Tumor volume of sh-circ group was lower than that of sh-NC group in mice (Fig. 8a). Tumor pictures were shown in Fig. 8b, and tumor weight was also deceased in sh-circ group contrasted to sh-NC group. The circ_0082182 level was reduced in sh-circ group by comparison with sh-NC group (Fig. 8c), showing the significant downregulation of circ_0082182 in mice. The knockdown of circ_0082182 then evoked the stimulative impacts on the miR-411 and miR-1205 levels (Fig. 8d). In addition, the silence of circ_0082182 repressed the protein levels of pGSK3β/GSK3β, Nucleus β-catenin, and N-cadherin but promoted the expression of E-cadherin (Fig. 8e). Altogether, circ_0082182 enhanced tumor growth and EMT process via the activation of Wnt/β-catenin pathway by acting as the sponges of miR-411 and miR-1205 in vivo.

## Discussion

The current research clarified the abnormal overexpression of circ_0082182 in CRC tissues and cells. The exploration of biological function and molecular mechanism has shown that circ_0082182 retarded CRC tumorigenesis and metastasis via activating the Wnt/β-catenin pathway by sponging miR-411 or miR-1205.

Increasing studies have highlighted the vital biological modulation of dysregulated circRNAs in various kinds of cancers. Liu et al. declared that hsa_circ_0102272 was notably upregulated in thyroid cancer, and it functioned as a prognostic indicator [31]. Our expression analysis indicated that circ_0082182 expression was increased in 73 CRC tissues and the overall survival was reduced in patients with high expression of circ_0082182, suggesting the diagnostic and prognostic values of circ_0082182 in CRC. Qiu et al. found that circ_103809 enhanced cell proliferation and cell cycle in breast cancer [32]. Circ_0007694 was identified to be downregulated in papillary thyroid carcinoma and inhibited tumor progression as a therapeutic target [33]. Circ_0082182 was also downregulated in CRC cells relative to normal cells. The current investigation on CRC cellular behaviors suggested that knockdown of circ_0082182 suppressed cell proliferation and cell cycle progression, but it led to the promotion of cell apoptosis. It was validated that circ_0082182 functioned as a carcinogenic molecule in CRC.

In addition to tumorigenesis, circRNA also has a pivotal effect on cancer metastasis [34]. It has been shown that some downregulated circRNAs were correlated to CRC with lung metastasis [35]. CircRNA_0001178 and circRNA_0000826 were considered as potential biomarkers to diagnose the liver metastasis from CRC [36]. Circ_0082182 downregulation was exhibited to trigger the inhibitory effects on migration and invasion in CRC cells. Moreover, EMT is a biological process in which epithelial cells acquire the migratory and aggressive abilities to possess the mesenchymal features [37, 38]. Through the determination of EMT-associated proteins (anti-EMT E-cadherin and pro-EMT N-cadherin), EMT process was presented to be impeded by circ_0082182 knockdown in CRC cells. Circ_0082182 could be used as a diagnostic target for CRC metastasis and its expression downregulation might inhibit the further metastasis of CRC.

In 2013, a circRNA (ciRS-7) was first identified as an efficient sponge for miR-7 [39]. Subsequently, numerous circRNAs have been indicated to generate the sponge effects on different miRNAs. For instance, hsa_circ_0000263 contributed to cell proliferation and migration in cervical cancer by for the sponge function for miR-150-5p [40]; circHMCU accelerated the proliferation and metastasis in breast cancer by sponging the let-7 miRNA family [41]; circ_0067934 facilitated the progression of laryngeal squamous cell cancer by sponging miR-1324 [42]. The sponge effects of circ_0082182 on miR-411 and miR-1205 were first confirmed in the present study. The functional assays for miR-411 and miR-1205 indicated that they acted as tumor repressors in CRC to inhibit cell proliferation, cell cycle, migration, invasion, EMT, and promote cell apoptosis. More importantly, miR-411 and miR-1205 inhibitors restored all these effects of circ_0082182 knockdown on CRC cells. The regulation of circ_0082182 in CRC progression was achieved by serving as the sponges of miR-411 and miR-1205, at least in part.

CircRNAs can affect tumor development via the modulation of Wnt/β-catenin signaling pathway [43]. For example, hsa_circ_0017247 [44] and circ-SOX4 [45] respectively worked as tumorigenic factors in bladder cancer and non-small cell lung cancer by activating the Wnt/β-catenin signaling pathway. Ma et al. asserted that hsa_circ_0005615 contributed to the malignant progression of CRC cells via the activation of Wnt/β-catenin pathway by sponging miR-149-5p [46]. β-catenin is a crucial downstream effector in the Wnt/β-catenin pathway and pGSK3β can prevent the β-catenin degradation to cause the accumulation of β-catenin in the nucleus to participate in various cell behaviors [47]. Our Western blot results indicated that silencing circ_0082182 decreased the levels of pGSK3β/GSK3β and nucleus β-catenin by increasing miR-411 and miR-1205, manifesting that circ_0082182 could activate the Wnt/β-catenin pathway via targeting miR-411 and miR-1205 in CRC cells. Furthermore, in vivo experiments also demonstrated that circ_0082182 promoted tumorigenesis and EMT process through the miR-411 or miR-1205-mediated activation of Wnt/β-catenin pathway.

This study has certain limitations. Firstly, the rescued experiments need to be performed in vivo to better confirm the circ_0082182/miR-411 and circ_0082182/miR-1205 axes. Secondly, other signaling pathways of circ_0082182 in CRC regulation by targeting miR-411 and miR-1205 remain to be explored. Last but not the least, the downstream target genes of miR-411 and miR-1205 need to be discovered. It is necessary to study whether circ_0082182 can regulate the gene expression to affect the Wnt/β-catenin pathway by acting as the sponges of miR-411 and miR-1205 in CRC progression.

## Conclusion

In conclusion, circ_0082182 was affirmed to promote the progression of CRC in vitro and in vivo by acting as the miR-411 and miR-1205 sponges to activate the Wnt/β-catenin signaling pathway. Circ_0082182 may serve as a diagnostic circRNA for CRC. More importantly, the inhibition of circ_0082182 expression may help to develop new therapeutic strategies for CRC.

## Supplementary Information

**Additional file 1: Supplementary Fig. 1.** Cellular pictures for Fig.5. (A) The pictures of colony formation for Fig.5c. (B) Cell cycle pictures for Fig.5d. (C) The apoptotic cell pictures for Fig.5e. (D-E) Cell migratory pictures for Fig.5g. (F) Cell invasive pictures for Fig.5h.

**Additional file 2: Supplementary Fig. 2.** Cellular images for Fig.6. (A) The images of colony formation for Fig.6c. (B) Cell cycle images for Fig.6d. (C) The images of apoptotic cells for Fig.6e. (D-E) The images of cell migration for Fig.6g. (F) The images of cell invasion for Fig.6h.

## Data Availability

Data sharing is not applicable to this article as no datasets were generated or analyzed during the current study.

## Abbreviations

CRC: Colorectal cancer
CCK-8: Cell counting Kit-8
EMT: Epithelial-mesenchymal transition
