# Supplementary figures and images for: Circ_0082182 promotes oncogenesis and metastasis of colorectal cancer in vitro and in vivo by sponging miR-411 and miR-1205 to activate the Wnt/β-catenin pathway
Source: World J Surg Oncol. 2021 Feb 17;19:51. doi: 10.1186/s12957-021-02164-y (PMC7891146; doi:10.1186/s12957-021-02164-y)

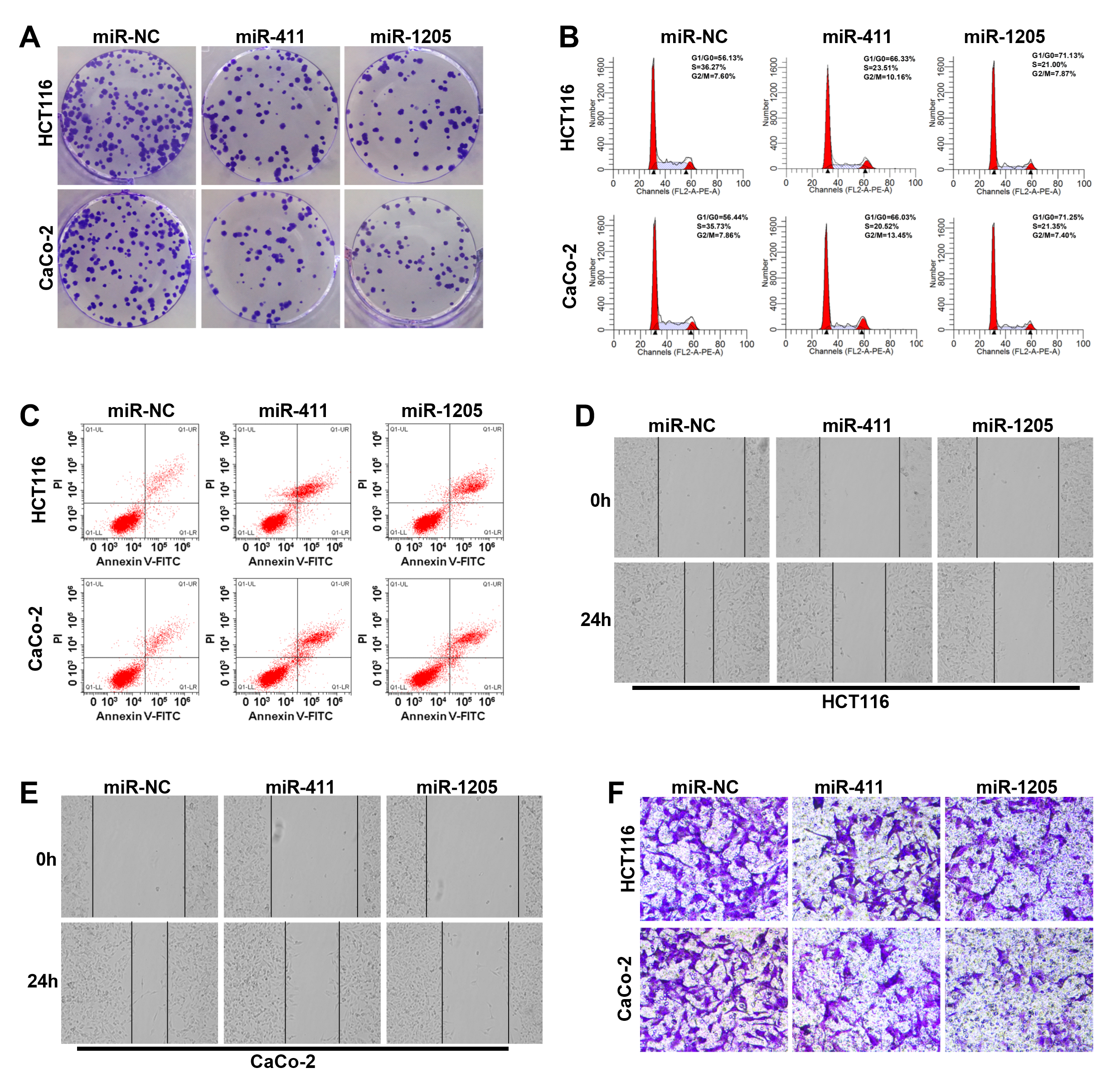

Supplement: Supplementary file 1 — Additional file 1: Supplementary Fig. 1. Cellular pictures for Fig.5. (A) The pictures of colony formation for Fig.5c. (B) Cell cycle pictures for Fig.5d. (C) The apoptotic cell pictures for Fig.5e. (D-E) Cell migratory pictures for Fig.5g. (F) Cell invasive pictures for Fig.5h. [file 12957_2021_2164_MOESM1_ESM.tif]

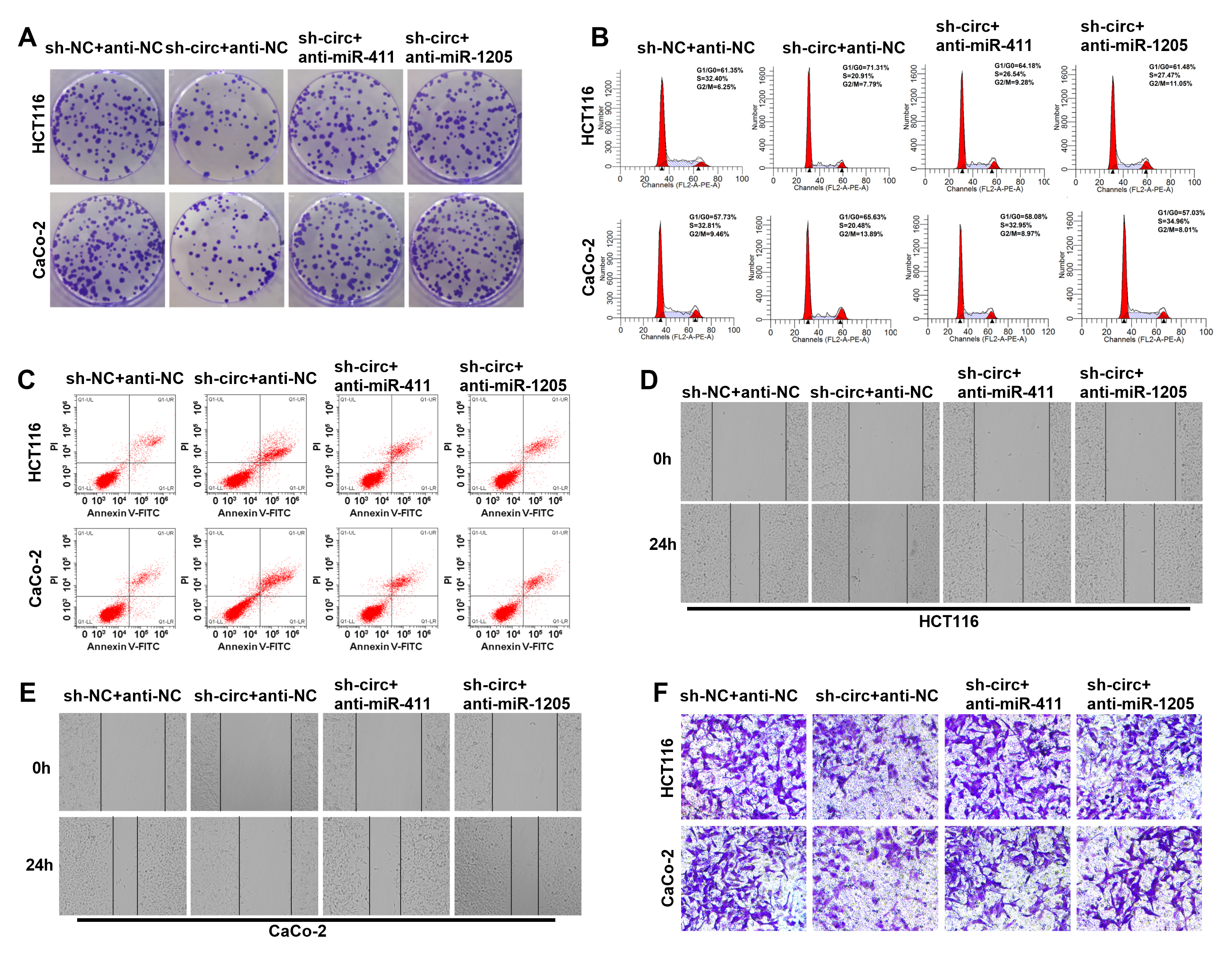

Supplement: Supplementary file 2 — Additional file 2: Supplementary Fig. 2. Cellular images for Fig.6. (A) The images of colony formation for Fig.6c. (B) Cell cycle images for Fig.6d. (C) The images of apoptotic cells for Fig.6e. (D-E) The images of cell migration for Fig.6g. (F) The images of cell invasion for Fig.6h. [file 12957_2021_2164_MOESM2_ESM.tif]
